# Editorial: Allergen cross-reactivity - a challenge in daily practice

**DOI:** 10.3389/falgy.2026.1846379

**Published:** 2026-04-21

**Authors:** Marcin Kurowski, Rosemarie DeKruyff, Daniela Briceno Noriega

**Affiliations:** 1 Department of Immunology and Allergy, Medical University of Lodz, Lodz, Poland; 2 Sean N. Parker Center for Allergy and Asthma Research, Department of Pathology, Stanford University School of Medicine, Stanford, CA, United States; 3 Wageningen University & Research, Wageningen, Netherlands

**Keywords:** airborne allergens, allergy, component-resolved diagnosis (CRD), cross-reactivity, drug allergy, food allergy

Cross-reactive reactions between allergens from apparently distant allergen sources pose considerable challenges in daily allergy practice. Spectacular examples of cross-reactivity include food-pollen allergy syndrome (FPAS) (1), latex-fruit syndrome (LFS) (2, 3), as well as cross-reactivity between dust mite and shrimp-derived tropomyosin (4–6). However, one can suspect that multiple apparently idiopathic or unexplained reactions might be triggered by cross-reactivity between allergenic components, the presence of which has not yet been ascertained and does not appear to be a possible cause of patients' symptoms. Spectacular progress in the field of component-resolved diagnostics (7) over the last few years has contributed to increased knowledge and awareness of cross-reactivity and to the discovery of new cross-sensitizations. These facts are of considerable significance for improving allergy management practices.

Considering the above, I am happy to introduce you to the Research Topic “*Allergen cross-reactivity - a challenge in daily practice*” which has been successfully published in the “Frontiers in Allergy”. Together with Rosemarie DeKruyff as a Co-editor and Daniela Briceno as a Topic Coordinator, we have collected contributions that cover various aspects of cross-reactive allergic reactions. This collection contains 2 original research articles, 2 reviews, and one case report, all of which align with the aforementioned aspects of less apparent and less obvious cross-sensitizations.

Reina et al. in their original research explored cross-sensitization and cross-reactivity between the allergens of house dust mites (HDM) and cockroaches in the tropical environment. Their results identify house dust mite allergens as primary sensitizers in inhabitants of a tropical environment of the city of Cartagena, Colombia. In addition, these authors have explored cross-sensitizations between HDM allergenic proteins and those of cockroaches, shrimp, and *Anisakis simplex*, concluding that cockroach sensitization should be considered as important hallmark of potential cross-reactivity with allergens coming from crustaceans, and, therefore, warrant more thorough approach during clinical assessment.

In the second original paper in this Research Topic, Maskey et al. approach, using the mouse model, an important clinical problem of cross-reactivity between peanut and tree nut allergens. In an attempt to study this potential cross-sensitization, an intraperitoneal sensitization (priming) with a mix of peanut, tree nuts and cashew allergens was performed, and subsequently, specific IgE levels against priming and potentially cross-reactive allergens were measured. Broad cross-reactivity was ascertained, as assessed through specific IgE levels, additionally confirmed by experimental anaphylaxis. Development of this model can be considered a valuable contribution to the advancement of the research on nut cross-reactivity.

This Research Topic also contains 2 review papers, which present topics valuable from both basic and clinical points of view. Berghea et al., in their narrative review, describe the wide spectrum of allergic sensitization to spices and herbs, with special focus on the pediatric population. Both IgE-dependent and non-IgE-dependent mechanisms have been highlighted, and the problem of cross-reactivity with pollen allergens was indicated. In the second review contained in this Topic, O'Malley et al. describe calcium-binding proteins (CBPs), an important yet often neglected family of allergenic proteins that, on a standard basis, is not considered a potential cause of cross-reactive reactions. Calcium-binding proteins, due to their heterogeneity, cross-reactivity, and presence in multiple allergenic sources, are difficult to avoid. The fact that CBPs can sensitize both through inhalation and ingestion makes them even more worth consideration in the course of a diagnostic approach in an allergy practice.

Finally, Schreiber and Guillet presented a description of a rare case of severe IgE-mediated hypersensitivity to carboxymethylcellulose (CMC) following an intraarticular knee injection with triamcinolone acetonide. A thorough diagnostic approach allowed them to diagnose a patient with IgE-mediated, and clinically relevant, hypersensitivity to CMC, yet with confirmed lack of sensitization to potentially cross-reactive cellulose derivatives. Reports describing rare cases of IgE-mediated generalized reactions to less obvious agents, such as excipients exemplified by CMC, largely contribute to expanding our knowledge and increase our vigilance and critical thinking. Such an approach is irreplaceable when aiming at highest levels of diagnostic insightfulness and thoroughness.

We would like to express our gratitude to all contributors to this Research Topic. It was a pleasure to be able to co-edit such an impressive set of contributions. We sincerely encourage the allergy community worldwide to delve into the fascinating area of possible (and also apparently impossible…) allergic cross-rectivities.
